# Oxidative Stress and X-ray Exposure Levels-Dependent Survival and Metabolic Changes in Murine HSPCs

**DOI:** 10.3390/antiox11010011

**Published:** 2021-12-22

**Authors:** Melis Karabulutoglu, Rosemary Finnon, Lourdes Cruz-Garcia, Mark A. Hill, Christophe Badie

**Affiliations:** 1 Cancer Mechanisms and Biomarkers Group, Radiation Effects Department, Radiation, Chemical and Environmental Hazards Directorate (RCE, Formally CRCE), UK Health Security Agency (Formerly Public Health England), Chilton, Didcot, Oxon OX11 0RQ, UK; rosemary.finnon@phe.gov.uk (R.F.); lourdes.cruzgarcia@phe.gov.uk (L.C.-G.); 2 MRC Oxford Institute for Radiation Oncology, Department of Oncology, University of Oxford, Oxford OX3 7DQ, UK; mark.hill@oncology.ox.ac.uk

**Keywords:** HSPCs, oxidative stress, ionising radiation, metabolism, radiation leukemogenesis, mitochondrial dysfunction, hypoxia, reactive oxygen species, acute myeloid leukaemia

## Abstract

Haematopoietic bone marrow cells are amongst the most sensitive to ionizing radiation (IR), initially resulting in cell death or genotoxicity that may later lead to leukaemia development, most frequently Acute Myeloid Leukaemia (AML). The target cells for radiation-induced Acute Myeloid Leukaemia (rAML) are believed to lie in the haematopoietic stem and progenitor cell (HSPC) compartment. Using the inbred strain CBA/Ca as a murine model of rAML, progress has been made in understanding the underlying mechanisms, characterisation of target cell population and responses to IR. Complex regulatory systems maintain haematopoietic homeostasis which may act to modulate the risk of rAML. However, little is currently known about the role of metabolic factors and diet in these regulatory systems and modification of the risk of AML development. This study characterises cellular proliferative and clonogenic potential as well as metabolic changes within murine HSPCs under oxidative stress and X-ray exposure. Ambient oxygen (normoxia; 20.8% O_2_) levels were found to increase irradiated HSPC-stress, stimulating proliferative activity compared to low oxygen (3% O_2_) levels. IR exposure has a negative influence on the proliferative capability of HSPCs in a dose-dependent manner (0–2 Gy) and this is more pronounced under a normoxic state. One Gy x-irradiated HSPCs cultured under normoxic conditions displayed a significant increase in oxygen consumption compared to those cultured under low O_2_ conditions and to unirradiated HSPCs. Furthermore, mitochondrial analyses revealed a significant increase in mitochondrial DNA (mtDNA) content, mitochondrial mass and membrane potential in a dose-dependent manner under normoxic conditions. Our results demonstrate that both IR and normoxia act as stressors for HSPCs, leading to significant metabolic deregulation and mitochondrial dysfunctionality which may affect long term risks such as leukaemia.

## 1. Introduction

Haematopoiesis relies on the fine regulation of bone marrow (BM) haematopoietic stem cell (HSC) biology in response to continuously changing internal and external cues from the niche microenvironment [1,2]. A functional link between stemness and reduced O_2_ availability has been stated in multiple stem cell contexts and has been extensively studied in the haematopoietic stem and progenitor cell (HSPC) population [3,4]. The low O_2_ environment of the BM endosteal stem cell niche supports the maintenance of long-term hematopoietic stem cells (LT-HSCs) in a protective quiescent state, whereas the actively differentiating/proliferating short-term HSCs (ST-HSCs) and progenitor cells are in the higher O_2_ environment of the perivascular niches [3,5,6,7,8,9].

Mitochondria are key organelles in cellular metabolism, generating adenosine-5’-triphosphate (ATP) via an electron transport chain (ETC) driven by substrate oxidation (known as oxidative phosphorylation (OXPHOS). Recent evidence indicates that mitochondria play a vital role in HSC quiescence and their ability to switch from dormancy into a metabolically active state [10,11,12]. LT-HSCs residing in a low O_2_ environment have a highly glycolytic nature with relatively low mitochondrial mass and activity, consistent with low Reactive Oxygen Species (ROS) levels [3,12,13,14,15,16,17,18]. Inversely, HSPCs with reduced self-renewal competence (e.g., ST-HSCs, Multipotent Progenitor Cells (MPPs)) generate their energy by shifting the metabolic state from anaerobic glycolysis to aerobic OXPHOS, increasing both mitochondrial mass and activity [19,20]. Adaptation of HSCs to differing O_2_ environments, therefore, depends on alterations to mitochondrial number, size and activity, which are controlled by de novo synthesis, degradation, fission and fusion processes [21,22,23]. Interference of any of these regulatory processes may lead to accumulation of dysfunctional or damaged mitochondria, impeding cellular function [24]. 

Metabolic equilibrium is critical for HSC maintenance, haematopoietic homeostasis and restraining the generation of ROS. Changes in redox status, induced by internal and external factors such as diet, can alter this equilibrium leaving haematopoiesis vulnerable to dysregulation/dysfunction [10,25]. Mitochondrial ETC is the main producer of ROS in the cell and accumulation of ROS-induced mitochondrial DNA mutations in HSCs can interrupt multi-lineage differentiation [15,26]. Likewise, aberrant increases in mitochondrial metabolism and function can trigger cell-cycle entry, resulting in a significant loss in adult HSC quiescence [27,28]. Even though ROS were primarily considered to be harmful by-products of metabolism, growing evidence disclosed the importance of moderate levels in cell-fate signalling, stem cell function and development in the bone marrow microenvironment [29,30,31,32].

The carcinogenic potential of ionizing radiation (IR) was recognised soon after Roentgen’s discovery of X-rays in 1895 and the first cases of leukaemia were suspected in medical radiation workers [33]. Epidemiological studies of the Japanese Atomic-bomb survivors revealed an increased risk of leukaemia with acute myeloid leukaemia (rAML) the most predominant radiation-associated leukaemia, accounting for around 80% of cases [34]. AML is an aggressive neoplasm characterised by a differentiation block in myelopoiesis which leads to a failure to produce mature myeloid (granulocytes) cells, and an over-proliferation of immature dysfunctional myeloid cells (leukemic or myeloblasts). The haematopoietic system is particularly radiosensitive due to being a regenerative tissue with a high proliferation potential. Ionising radiation has multiple effects on HSCs, from induction of oncogenic mutations to long-lasting reductions in the number of Lin^-^ Sca-1^+^cKit^+^ (LSK) cells. Such damage can lead to long term functional defects and transformation; therefore, it is a tissue at risk of radiation-induced leukemogenesis.

Murine radiation-induced AML (rAML) models have identified the cytogenetic and molecular alterations leading to rAML and provided further insight into the genetic basis of human radiation leukemogenesis [35,36]. Bi-allelic mutations of the haematopoietic regulator gene Spi1 are a common feature of murine rAML, with a large hemizygous deletion (known as del2) removing one copy, and a point mutation (known as R235) in the other [37,38,39]. Del2 is an early event, detectable at 24 h post-irradiation in bone marrow cells [40,41]. Recently, our group used a F1 CBA *Spi1 ^Gfp/mCherry^* reporter mouse [36] to detect expanding clones of del2-positive leukocytes in the peripheral blood. Prevalence of del2 clones increased with time, being detectable in 25% and >50% of mice by 9- and 12-months post-irradiation respectively. Despite this, the rAML presentation rate is only 15–20%, with a long latency (around 14 months), suggesting that whilst del2-clones appear to have a growth advantage, this is insufficient for full AML presentation, and acquisition of the R235 point mutation and/or other secondary events at some later stage is required. Whilst a clear link between radiation and leukaemia development is acknowledged, understanding of the underlying mechanisms remains incomplete. The identity and characteristics of the target cell for rAML within the HSPC compartment and the timing of R235 occurrence post-irradiation in del2-carrying HSPC are unknown. In addition, further investigation into secondary events and internal and external risk factors for rAML susceptibility e.g., inherited genetic and epigenetic alterations, diet and metabolism, is required.

Exposure to IR can amend metabolic activity, provoke ROS generating-oxidases, and modulate antioxidants in response to oxidative stress. Normoxia is known to enhance IR-induced oxidative DNA damage whereas low O_2_ environments are more radio-protective and hypoxia in tumours can lead to radio-therapy resistance. Additionally, DNA damage profiles and subsequent DNA repair pathways can vary between differing O_2_ environments [42,43]. Mitochondria are known to be particularly radio-sensitive as mitochondrial DNA (mtDNA) has no protective histones and mitochondria have limited DNA repair capacity and so IR-exposure could lead to accumulation of mutations in mtDNA, priming OXPHOS impairment and mitochondrial dysfunctionality [44,45,46].

Diet and energy metabolism influence the regulation of tissue stem cells and their homeostasis to alter tissue composition and growth, indicating interconnection between diet, stem cell function and cancer/leukaemia development and a relationship between metabolism and cancer development at the cellular level has been long acknowledged [47]. Epidemiology has identified that many human malignancies are attributable to diet and metabolism [48], and obesity and insufficient physical activity are known to increase the risk of many cancers [49] including leukaemia [50,51]. What is being revealed is an intricate network of dietary and metabolic factors which interact to form a complex metabolic regulatory system maintaining haematopoietic homeostasis and may act to modulate stem cell radiosensitivity and rAML risk [52]. However, the effect of dietary alterations such as calorie restriction, fasting, depletion of specific nutrients (e.g., individual amino acids, vitamins) and other metabolic changes on HSC regulatory systems and leukaemia risk requires further understanding and represents a significant research area of interest to both the Radiation Protection and Public Health fields.

Therefore, we have begun to explore the underlying mechanisms to better understand the role of O_2_ metabolism on HSC function and radiation-induced leukemogenesis. In this study we describe using the Seahorse XFp Analyzer to assess the effect of IR exposure on mitochondrial function and O_2_ metabolism in primary HSPC cultures obtained from a rAML-sensitive CBA/Ca mouse model under normoxic (20.8%) and low (3%) O_2_ conditions. The data produced from this model system will form the basis for future studies to identify dietary factors which may mitigate the negative effects of IR exposure and modify rAML risk.

## 2. Materials and Methods

### 2.1. Mice

Mice from a CBA Spi1^Mcherry^ breeding colony based at UK Health Security Agency Radiation, Chemical and Environmental Hazards Directorate (Harwell, UK) was used as a source of haematopoietic tissue for HSPCs [36]. All animals were bred and handled according to the UK Animals (Scientific Procedures) Act 1986, Amendment Regulations 2012. Animal protocols were reviewed and approved by the local Ethics Committee and the 83 Home Office.

### 2.2. X-ray Exposures

In vitro irradiations (0.1–2.0 Gy) were performed at PHE CRCE using an AGO X-ray set (AGO, Reading, UK) running at 250 kV (constant potential) with a Cu/Al filter producing a beam of 1.2 mmCu HVL, with a high dose rate 0.5 Gy/min (13 mA, source to shelf distance of 60 cm) or low dose rate 4.9 mGy/min (0.2 mA, source to shelf distance of 91 cm) and filtration remained the same under all circumstances. All exposures were performed at room temperature and under normoxia (20.8% O_2_).

### 2.3. Tissue Harvest and Immunogenic Negative Selection of HSPCs

Mice were sacrificed with a rising concentration of CO_2_ and tibias, femur, iliac crests, and spine dissected and cleaned of remaining muscle and connective tissue. To extract Bone Marrow (BM) cells the cleaned bones were crushed in a pestle and mortar in a small volume of Iscove’s Modified Dulbecco’s Media (IMDM) and a single cell suspension generated by disaggregating with an 18 G needle and filtering through a 40 µm cell strainer (BD Biosciences, Wokingham, UK) into a centrifuge tube. The cells were centrifuged for 5 min at 1200 rpm and the pellet resuspended in 1 mL IMDM ready for use.

For immunomagnetic selection of HSPCs, BM cell numbers were determined using a Neubauer haemocytometer and cell densities were adjusted at a concentration of 1 × 10^8^ cells/mL in RoboSep^TM^ Buffer in a 14 mL polystyrene round-bottom tube (Thermo Fisher Scientific, Loughborough, UK) and 50 μL/mL of Normal Rat Serum added, and the tube placed in the RoboSep machine. Fully Automated Mouse hematopoietic progenitor cell isolation protocol #catalogno 19856 (Stem Cell Technologies, Cambridge, UK) was selected, the machine was loaded with antibody cocktail, RapidSpheres^TM^, RoboSep^TM^ buffer and RoboSep^TM^ filter tips, following on-screen instructions. All reagents were obtained from Stem Cell Technologies, unless otherwise stated.

### 2.4. Culture Conditions for HSPC Expansion

Following immunogenic negative selection, HSPCs were seeded at the required concentration in a specialised StemSpan (SSpan)^TM^ and a custom-made (amino acid-free) StemSpan^TM^ serum-free expansion medium supplemented with 50 ng/mL recombinant murine stem cell factor, 100 ng/mL recombinant human Flt3 ligand, 100 ng/mL recombinant human interleukin 11, 40 μg/mL low density lipoprotein (Sigma-Aldrich; Invitrogen, Alfa Aesar, Lancashire, UK), 100 U/mL penicillin, 100 μg/mL streptomycin (Thermo Fisher Scientific) and 50 mM 2-mercaptoethanol (Gibco, Life Technologies, Loughborough, UK) in 6-well plates and incubated at 37 °C, 5% CO_2_. Cultures were set up in triplicate wells and experiments were performed in triplicate per dose.

For amino acid depletion (Section 3.2.3), a stock of non-essential amino acid mix and aliquots of 16 essential amino acids (arginine·HCl, arginine, cystine·2HCl, cysteine·HCl· H_2_O, glutamine, histidine ·HCl·H_2_O, hydroxyl proline, isoleucine, leucine, lysine·HCl, methionine, phenylalanine, threonine, tryptophan, tyrosine ·2Na·2H_2_O and valine, all L-isomers; Sigma-Aldrich, Gillingham, UK) were added individually to yield variety of individual media deficient in single amino acids.

### 2.5. HSPC Proliferation Assay

HSPCs were plated at a concentration of 2 × 10^5^ cells/mL in Stemspan medium into a 6-well tissue culture plate and incubated at 37 °C either in a 20.8% or a 3% O_2_ incubator for 2 h. After 2 h of incubation, cells were removed from the incubator and exposed to different radiation doses (0.1–2.0 Gy) prior to being incubated at 37 °C either in a 20.8% or a 3% O_2_ incubator. Growth rates were examined, counting the number of HSPCs on days 2, 5, 7, 9, 13, 15 and the average number of cells were represented by generating a growth curve. One mouse per sample was used unless otherwise stated and all experiments were performed in triplicates.

### 2.6. Seahorse XFp Assay for Assessment of Energy Metabolism

Initially, the optimal seeding density was evaluated, using a range of cell numbers from 1 × 10^4^, 1 × 10^5^ or 2 × 10^5^ cells per well. 2 × 10^5^ cells/well was selected as an optimal seeding density for a consistent confluent monolayer. The Agilent Seahorse XFp Analyzer was turned on overnight to warm up and the sensor cartridge was hydrated in Seahorse XF Calibrant (Agilent) following the manufacturer’s instructions (Agilent).

Agilent Seahorse cell culture miniplates were coated beforehand, using Cell-Tak solution (22.4 μg/mL). For each assay, 0.25 mL of Cell-Tak solution was made per plate, as per manufacturer’s instructions (Agilent Technologies, Didcot, UK) and 25 μL was applied to each well for 20 min at room temperature. Wells were washed twice using 200 μL distilled water and stored at 4 °C. On the day of the assay, HSPCs were harvested from the expansion cultures at day 4, 7 and 11 and plated into pre-warmed Cell-Tak coated miniplates in 180 µL XF base medium (DMEM non-buffered pH 7.4) supplemented with 200 mM glutamine, 1 M glucose and 100 mM pyruvate. For adherence, cells were spun down at 200× *g* for 2 min and incubated in a humidified non-CO_2_ incubator at 37 °C for 1 h before the assay.

For ATP-rate analysis, pre-warmed oligomycin (1.5 µM final), Rotenone and Antimycin A (0.5 µM final) were diluted in the assay medium and loaded into ports A or B of the hydrated sensor cartridge. For Cell Energy Phenotype Assay, a stressor mixture was prepared with diluted oligomycin (1.0 µM final) and FCCP (1.0 µM final) and loaded into port A, respectively. After calibration of the sensor cartridge, the Seahorse XF cell culture miniplate was inserted (Seahorse Bioscience, Billerica, MA, USA), and the assay continued either using Real-time ATP Rate or Cell Energy Phenotype protocols. Oxygen Consumption Rate (OCR) and Extracellular Acidification Rate (ECAR) were taken over time under basal conditions and after the addition of mitochondrial inhibitors. With the simultaneous injection of these stressor reagents two events proceeded: Oligomycin impeded mitochondrial ATP production and, consequently, there was a compensatory surge in glycolytic rate. FCCP depolarized the mitochondrial membrane which triggered the OCR to preserve the mitochondrial membrane potential. For data analysis, the XFp report generator programme was used automatically to calculate test parameters from Wave software. In all experiments three replicate wells were used. Respiration and acidification rates were presented as the mean ± SEM. All consumables were purchased from Seahorse Bioscience Inc. (North Billerica, MA, USA) (Agilent).

### 2.7. Mitochondrial Functionality Assessment

Following lineage depletion, HSPCs were expanded in suspension cultures at different oxygen states and X-irradiation exposures, as described in Section 2.3.

#### 2.7.1. Mitochondrial Mass

HSPCS were harvested from expansion culture at day 7, resuspended in Advanced RPMI 1640 (1 × 10^6^ cells/mL) and stained with 100 nM MitoTracker^®^ Deep Red FM (Thermo Fisher Scientific) dissolved in DMSO at 37 °C/5% CO_2_ for 30 min as per the manufacturer’s guidelines. Cells were then washed in prewarmed dPBS, resuspended in Advanced RPMI and stored on ice until being examined using a Guava^®^ easyCyte Single Sample Flow cytometer and analysed using InCyteTM software (Merck Millipore, Watford, UK).

#### 2.7.2. Mitochondrial Membrane Potential

HSPCs were harvested from expansion cultures at day 7, resuspended at 1 × 10^6^ cells/mL in RoboSepTM Buffer and incubated with 2 µM MitoProbeTM JC-1 cationic dye (Thermo Fisher Scientific; dissolved in DMSO) at 37 °C/5% CO_2_ for 30 min as per manufacturer’s guidelines. For control sample, 50 µM carbonyl cyanide 3-chlorophenylhydrazone (CCCP; Thermo Fisher Scientific) was added simultaneously. Cells were washed, resuspended in RoboSepTM Buffer, and stored on ice until analysis. Samples were analysed on a Guava^®^ easyCyte Single Sample flow cytometer, using 488 nm excitation with 530/590 nm emission filters. JC-1 is a lipophilic, cationic dye that can selectively enter mitochondria and reversibly change colour from green to red as the membrane potential increases. In healthy cells with high mitochondrial (ΔψM), JC-1 instinctively generates complexes known as J-aggregates (oligomers) with red fluorescence. Whereas, in unhealthy or apoptotic cells with low (ΔψM), JC-1 maintains its monomeric form with a green fluorescence. 

#### 2.7.3. Mitochondrial Superoxide

HSPCs were harvested from expansion cultures at day 7. Cells were resuspended at 1x10^6^ cells/mL in RoboSepTM Buffer and incubated with 5 µM MitoSoxTM Red (Thermo Fisher Scientific; dissolved in DMSO) at 37 °C/5% CO_2_ for 30 min as per manufacturer’s instructions. After incubations, cells were washed, resuspended in 0.5 mL RoboSepTM Buffer and stored on ice until being examined using a Guava^®^ easyCyte Single Sample flow cytometer and analysed using InCyteTM software (Merck Millipore, Watford, UK).

#### 2.7.4. Mitochondrial DNA Copy Number Assay

The ratio of mitochondrial DNA (mtDNA) to genomic DNA (gDNA) in HSPCs was measured using Quantitative Real-time Polymerase chain reaction (qRT-PCR) to quantitatively assess the ratio of genes on the mitochondrial and nuclear genome. To amplify the mitochondrial gene; Mus musculus mitochondrion, complete genome (mtND1: NCBI Reference number NC_005089.1) and the genomic gene; Mus musculus glyceraldehyde-3-phosphate dehydrogenase (GAPDH: NCBI Reference number NM_001289726.1), primers for both genes were designed using the online Primer3Plus software, obtained from Integrated DNA technologies and primer sequences can be found in Table 1. Total DNA (mtDNA and gDNA) was extracted from cells, using DNeasy Blood and Tissue Kit: Spin-Column Protocol (Qiagen) by following the manufacturer’s guidelines.

QRT-PCR was performed using a Rotor-Gene Q (Qiagen) with PerfeCTa SYBR^®^ Green SuperMix (Quanta Biosciences). The samples were run in triplicates in 10 µL reactions with 1 µL of the DNA sample (1 ng/µL) together with primer sets for mtND1and GAPDH (Table 1). Reactions were performed with the following cycling conditions: 2 min at 95 °C, then 40 cycles of 10 s at 95 °C (denaturation), 60 s at 60 °C (annealing/elongation). Fluorescence data acquired during the extension phase were normalized to the housekeeping gene GAPDH by the delta-delta method [53].

### 2.8. Hydrogen Peroxide Detection

Haematopoietic stem and progenitor cells were harvested from expansion cultures at day 7 and centrifuged at 1200× *g* for 5 min. Three hundred microlitres of supernatant (free of debris/cells) from each condition (three wells/condition) was transferred into 0.5 ml Eppendorf tubes and stored at −200 °C until analysis. Quantitative measurement of hydrogen peroxide was ensued by using a hydrogen peroxide microsensor probe; Figure S1a (World Precision Instruments).

The settings of the free radical analyser for H_2_O_2_ were adjusted. 20 mL of PBS (Solution #1) was measured and placed into a vial that would hold it. Then a small stirring bar was dropped into the solution and the vial was placed on top of a magnetic stirring plate. The RPM on the stirring plate was adjusted high enough to rapidly disperse aliquots of 1 mM H_2_O_2_ (Solution #2) throughout the buffer. The sensor was polarized overnight in PBS and the poise voltage was set at 400 mV. Background current was adjusted to a stable baseline value (1000 pA or less) with the free radical analyser set in the 10 nA range. For calibration, various ranges of aliquots (2 µL, 4 µL, 8 µL) of Solution #2 were added into the 20 mL vial containing the 20 mL of 0.1 M PBS buffer and output was observed after each addition. Each aliquot corresponded to the calculated concentrations of 100 nM, 200 nM and 400 nM respectively. Then, a calibration curve was generated by plotting the changes in the current (pA) against the changes in concentration (nM). Following successful calibration, 100 µL of media from each condition was added into the vial, voltage measurements were documented, and concentrations were calculated, using the standard curve. All reagents were obtained from Sigma-Aldrich, unless otherwise stated.

### 2.9. Statistical Analysis

GraphPad Prism 7.04 was used for statistical analyses. An assessment of normality was evaluated by applying the D’Agostino and Pearson analysis. Comparison between the two groups tested was performed using Student’s t-test, and between more than two groups by analysis of variance (ANOVA) and Dunn’s multiple comparison test. All data was expressed as mean values ± Standard Error of the Mean (±SEM).

## 3. Results

### 3.1. Effect of Ionising Radiation and Oxidative Stress on the Proliferative Capability of HSPCs

#### Growth Curve Analysis of HSPC Expansion Cultures Exposed to IR and Differing Oxygen Levels

**Figure 1:**
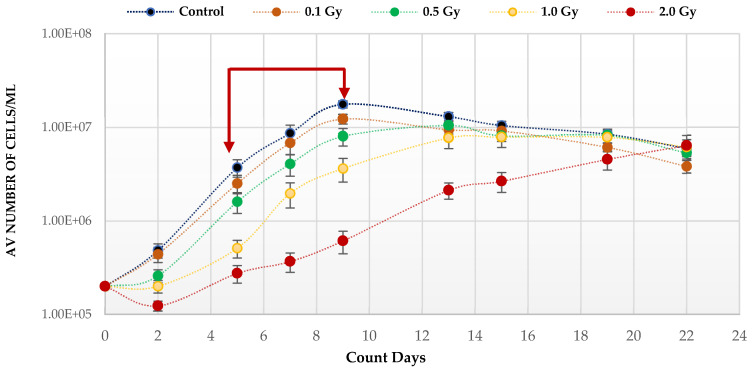
Proliferative capability of HSPCs following IR exposure under low (3%) O_2_ state. HSPCs (2 × 10^5^ cells/well, x-irradiated (0–2 Gy)) were incubated in 3% O_2_ and regular cell counts carried out to day 22 (*N* = 3/dose, 3 wells/dose/experiment. Error bars: ±SEM). Two-way ANOVA and Dunnett’s multiple comparison test compared IR- and non-IR exposed HSPCs (0.1 Gy *p <* 0.001; 0.5 Gy *p* < 0.001; 1.0 Gy *p* < 0.0001; 2.0 Gy *p*< 0.0001). Inter-dose comparisons showed significant difference in all scenarios (student t-test *p* < 0.05, *p* < 0.005). Red arrows indicate exponential growth phase (days 5–9). Time and dose showed statistical significance, but subject did not.

Quiescent HSC maintenance is vulnerable to changes in redox state in the hypoxic BM niche. To investigate the effect of IR, known to produce ROS, on the growth potential of HSPCs under low O_2_, cells were placed in expansion cultures, irradiated in vitro, and counted every 3–4 days up to Day 22. The growth curve analysis under low O_2_ (Figure 1) shows a statistically significant reduction on the growth rates post-irradiation compared to unirradiated, with the effect increasing with dose (*p* values; 0.1 Gy < 0.001; 0.5 Gy < 0.001; 1.0 Gy < 0.0001; 2.0 Gy < 0.0001). This is particularly marked after exposure to the highest 2 Gy dose. This effect on the growth rate is due to radiation induced cytotoxicity leading to an initial drop in cell number at day 2 post-irradiation. Surviving cells continued to divide after exposure and all cultures eventually reached a plateau (stationary phase) with a similar number of cells irrespective of dose and growth rate, due to the limitation of substrate available.

**Figure 2:**
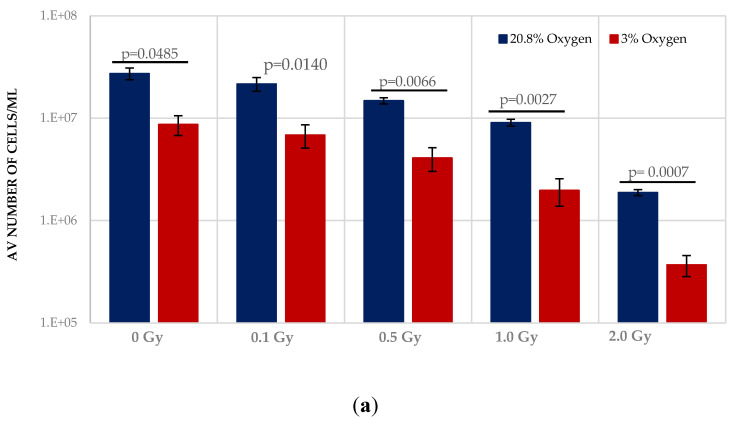

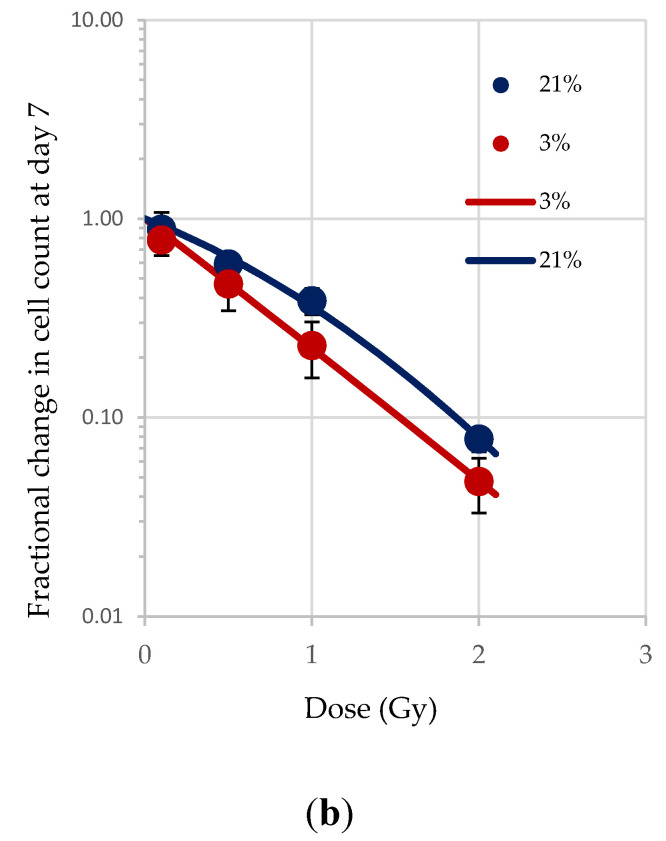
(**a**,**b**). Effect of oxygen stress and IR exposure on the proliferative capability of HSPCs at day 7. HSPCs (2 × 10^5^ cells/well, x-irradiated (0–2 Gy) were irradiated under normoxia and subsequently incubated under low O_2_ or normoxia and regular cell counts were carried out to day 14 (*N* = 3; 3 wells/dose/experiment; error bars represent ±SEM). ANOVA and multiple comparison tests were applied. Time and O_2_ level showed statistical significance in each dose with *p =* 0.0001 or *p <* 0.0001. Subject was not significant. The plating efficiency for both normoxic and low O_2_ cultures was ~75–80%.

Normoxia (20.8% O_2_) changes the redox state, favouring aerobic OXPHOS respiration over anaerobic glycolytic respiration. To determine the effect of this on HSPCs we compared the growth potential of irradiated HSPCs cultured in either low O_2_ or normoxic conditions (Figure 2). Significantly higher proliferative activity during exponential growth phase (between days 5–9 indicated by the red arrows in Figure 1) is seen in IR- and Non-IR exposed HSPC cultured in normoxic conditions compared with low O_2_ conditions (Figure 2a, day 7 cell counts representative of mid-point in exponential growth phase). A dose-dependent reduction in growth rate is seen with both normoxic and low O_2_ conditions but this effect is more pronounced in normoxia (*p* < 0.0001 Figure 2b), with a higher fractional change in day 7 cell count.

### 3.2. Effect of Radiation Exposure and Oxidative Stress on Mitochondrial Metabolism, DNA Content and Function

#### 3.2.1. Radiation Exposure and Oxidative Stress Influence Energy Metabolism

The Seahorse XFp analyser monitors changes in energy metabolism in cells by evaluating numerous bioenergetic parameters e.g., Mitochondrial Oxygen Consumption Rate (OCR), Extracellular Acidification Rate (ECAR; a measure of the rate of glycolysis) and Cellular Energy Phenotype assay. We have shown that both normoxia and IR exposure alter the growth potential of HSPCs, which are known to be affected by changes in redox state. To further understand this link between IR exposure, oxidative stress, and cellular/mitochondrial metabolism we used a Seahorse XFp analyser to study HSPC energy metabolism in low O_2_ and normoxic culture conditions and with increasing radiation dose (0–1 Gy).

**Figure 3:**
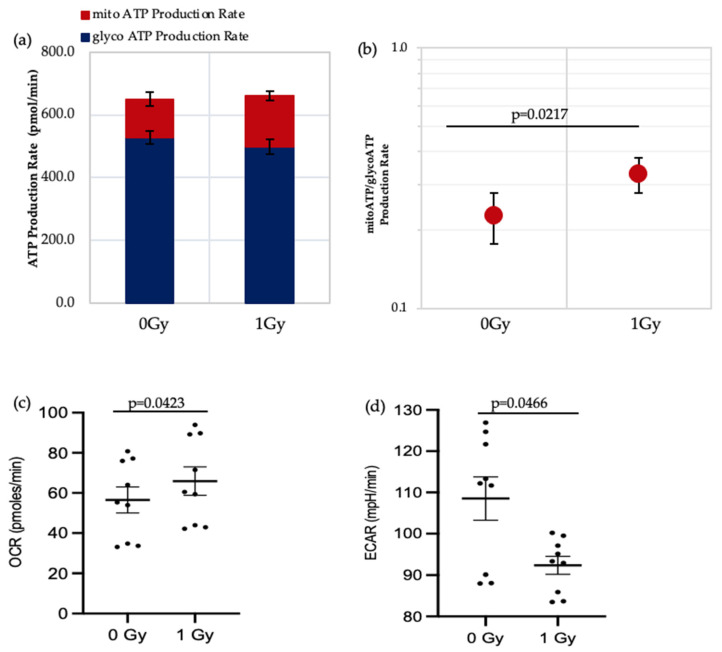
Seahorse XF real-time ATP rate analysis of HSPCs under low O_2_ environment. (**a**) Metabolic flux analysis shows increased OXPHOS when HSPCs are exposed to 1 Gy. (**b**) XF ATP rate index calculated from the data in panel a. (**c**,**d**) OCR: oxygen consumption rate. ECAR: extracellular acidification rate. Student *t*-test applied. Horizontal lines indicate statistical comparison made. Three independent experiments proceeded (*N* = 3; 3 wells/dose/experiment; error bars represent ±SEM).

ATP real-time assays synchronously measure ATP production in the mitochondrial and glycolytic pathways and so can be used to identify the favoured metabolic pathways of cells under varying conditions. We applied this assay to IR- and non-IR exposed HSPC in a low O_2_ environment (Figure 3a–d).

The data shows that there is a significant increase in the mitochondrial-ATP production rate (OXPHOS) in IR-exposed HSPC (Figure 3b,c), indicating that these cells switch to a more aerobic phenotype, whereas the non-IR exposed HSPCs favour an anaerobic glycolytic pathway (Figure 3a glycoATP production comparison between 0 Gy vs. 1 Gy, *p* = 0.006). HSCs residing in the hypoxic stem cell niche rely on anaerobic glycolysis which helps maintain quiescence and stemness [3,14,19]. Our results show increasing use of non-glycolytic metabolism in IR-exposed HSCs which can cause a switch from quiescence to a proliferating state and loss of stemness.

To give a more comprehensive picture of changing energy metabolism in HSPC in response to differing conditions we carried out XF cellular energy phenotype tests to determine the change in metabolic potential of IR- and non-IR exposed HSPC in normoxic or low O_2_ environments when stress conditions are applied using a stressor compound as part of the assay (methodological stressor compounds). Metabolic potential is the cell’s ability to meet an energy demand induced by a stressor and is defined by the difference between baseline and stressed OCR/ECR. The cellular energy phenotype test uses oligomycin (ATP-synthase inhibitor, which causes an increase in glycolysis) and FCCP (mitochondrial-uncoupling agent, increases oxygen consumption rates) as methodological stressor compounds.

**Figure 4:**
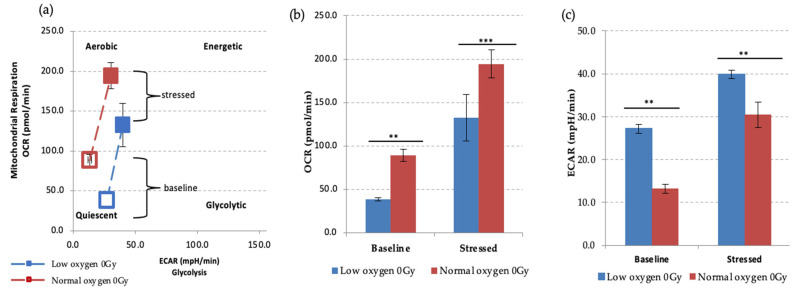
Seahorse XF Cell Energy Phenotype Test. (**a**) The phenotype map of HSPCs under basal and stress states. Non-IR exposed HSPCs were harvested from day 7 expansion cultures (*N* = 3, error bars represent ±SEM) (**b**,**c**) Basal and stressed OCR and ECAR measurements in HSPC under normoxic and low O_2_ conditions. Student t-test was applied to compare OCR (low oxygen vs. normal oxygen) and ECAR (low oxygen vs. normal oxygen). Asterisk * represents the significance difference; ** *p <* 0.05, *** *p <* 0.005.

We first examined the effect of different oxygen states on the metabolic potential of non-IR exposed HSPCs under baseline and stress conditions (Figure 4). Both under normoxic and low O_2_ environments HSPC populations showed significant increases of metabolic response with respect to baseline when methodological stressor compounds were applied (Figure 4a–c). However, in normoxia this is seen as increased mitochondrial oxygen consumption indicating an aerobic metabolic pathway, whereas low O_2_ favours a glycolytic pathway, as seen in the XF real-time ATP rate assay described previously.

**Figure 5:**
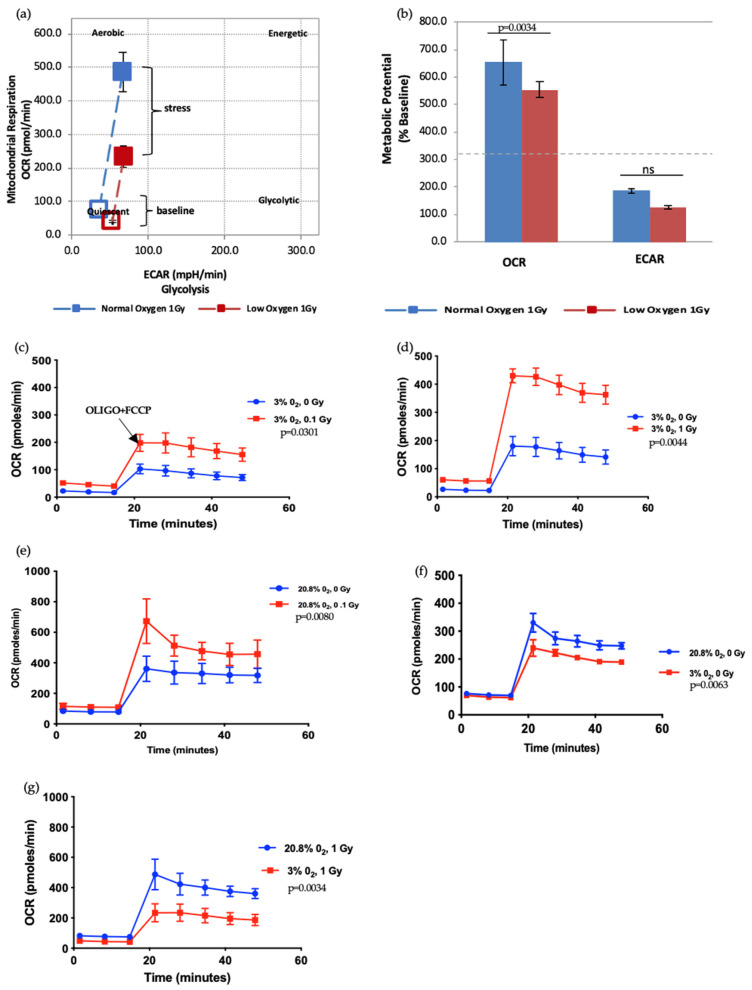
Seahorse XF Cell Energy Phenotype Test. (**a**,**b**) The phenotype map of 1 Gy exposed-HSPCs under normoxic and low O_2_ environments. HSPCs were harvested from day 7 expansion cultures (*N* = 3; error bars represent ±SEM). (**c**–**g**) Effect of different oxygen levels and radiation exposure on the metabolic potential of HSPCs. Student *t*-test applied for statistical analysis, *p* values are indicated separately for each figure.

Next, we further assessed the effect of radiation doses and oxygen status on the metabolic potential of HSPCs (Figure 5). HSPC exposed to a 1 Gy-IR exposure and kept in normoxia conditions showed higher mitochondrial respiration under baseline conditions. Likewise, when methodological stressors were added (black arrow marks the time-point when stressors added, in Figure 5c), these cells displayed more pronounced OCR when compared with 1 Gy-exposed cells under low O_2_ (Figure 5a). Conversely, there was no difference in ECAR between normoxia and low O_2_ (Figure 5b). This assay revealed that IR exposure also increases metabolic response in a dose-dependent manner (Figure 5c–g). Oxidative stress (normoxia) and increasing radiation exposure led to a pronounced increase in mitochondria-dependent ATP production to meet their energy demands under methodological stress conditions. Conversely, ECAR did not significantly alter (data not presented). IR exposure and normoxia both appear to act as physical oxidative stressors on HSPC, leading to increased mitochondrial ATP production, which suggests they may induce mitochondrial dysfunction.

#### 3.2.2. Effect of Ionising Radiation Exposure and Oxygen Level on Mitochondrial Mass, DNA Content and Function

**Figure 6:**
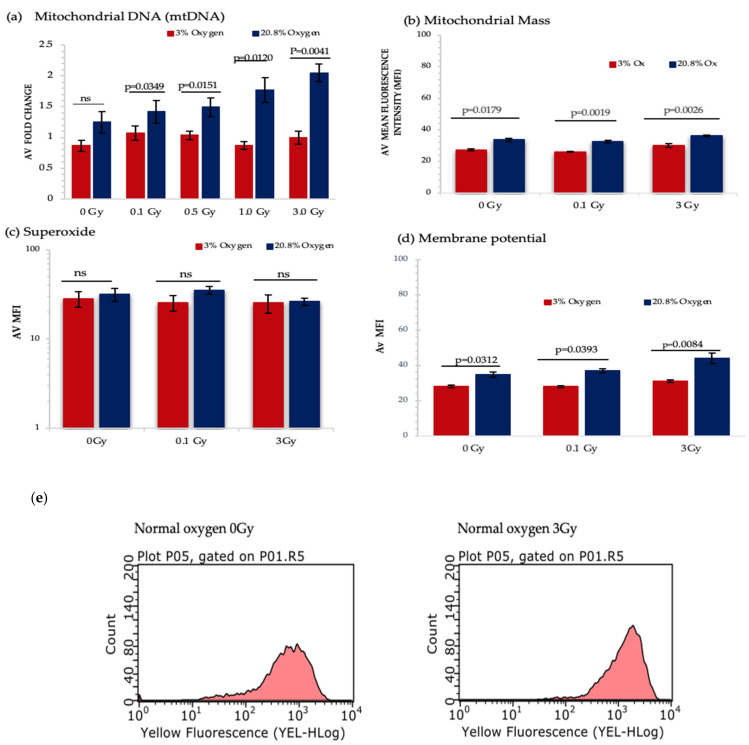
Represents the analysis from different mitochondrial functionality assays. HSPCs for each assay were extracted from expansion cultures at day 7. Different oxygen levels and radiation doses were compared to assess the influence on mitochondrial function. (**a**) Mitochondrial DNA-*N*3; (**b**) Mitochondrial Mass-*N*3, (**c**) Mitochondrial Superoxide-*N*4, (**d**) Mitochondrial Membrane Potential- *N*4. (**e**) Flow cytometric profiles of JC-1 displaying mean fluorescence intensity of non-IR and 3 Gy-exposed HSPCs under normoxia. Error bars represent ±SEM. ANOVA and multiple comparison test applied.

Our results so far have identified that normoxia and IR act as metabolic stressors in HSPC causing increased use of aerobic mitochondria-mediated respiration, leading to increased ROS generation, and therefore increasing the risk of mitochondrial dysfunction. To investigate this potential mechanism further we evaluated the effect produced by IR exposure and differing oxygen level conditions on indicators of mitochondrial function, in particular mitochondrial mass, DNA content, superoxide production and membrane potential (Figure 6a–d).

A significant increase in mitochondrial DNA (mtDNA) content was detected under normoxia and IR exposure in a dose dependent manner, whereas IR exposure did not produce any change in mtDNA content under low O_2_ background (Figure 6a). However, IR exposure did not produce any significant change in mitochondrial mass, whereas normoxia triggered a remarkable rise in mitochondrial mass when compared with a low O_2_ state (Figure 6b).

Mitochondrial ETC creates an electrochemical gradient which involves in ATP synthesis [54] and produces the mitochondrial membrane potential (MtMP), which is a key parameter for assessing mitochondrial function and is used as an indicator of cell health [55]. Our results show a substantial increase in MtMP under normoxia for all three doses. Furthermore, significant change in MtMP was detected between 0 Gy vs. 3 Gy and 0.1 Gy vs. 3 Gy-exposed HSPC population both under normoxic and low O_2_ states (Figure 6d).

Using the MitoSox assay there was no significant difference seen in superoxide levels under different oxygen states and IR exposure (Figure 6c). This may be due to the transient nature of ROS meaning that after 7 days in culture none remained detectable. Additionally, MitoSox only detects superoxide and does not identify other ROS and reactive nitrogen species (RNS). To begin to address this we decided to measure the level of hydrogen peroxide (H_2_O_2_) produced which is derived from superoxide. According to our preliminary data under a normoxic environment, there is a significant increase in H_2_O_2_ levels between 0 Gy vs. 3 Gy and 0.1 Gy vs. 3 Gy-exposed HSPCs (*p* < 0.0001; Figure S1b).

#### 3.2.3. Effect of Amino Acid Depletion on HSPCs Growth Rates and Metabolism

Recent studies identified the importance of AA metabolism in HSC maintenance and function [3,17,56,57] where HSCs cultured in individual AA-deprived conditions showed valine, methionine, and threonine (VMT) as essential for maintaining primitive HSCs. In addition, dietary-valine restriction has been shown to empty the mouse BM niche, affording donor HSC engraftment without a need for chemoirradiative myeloablation [58]. Kornberg et al. identified that HSPCs are particularly sensitive to protein deprivation, and that there is distinct enrichment of soluble AAs within the BM when compared with peripheral blood [59]. Moreover, in vitro growth studies identified that valine (and to a lower extent cysteine) are essential for maintaining mouse HSC survival and proliferation [57,58]. 

**Figure 7:**
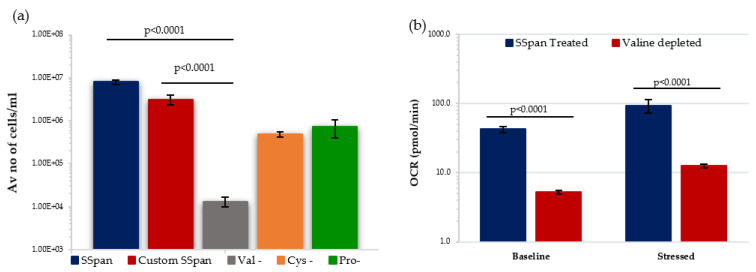
Effect of amino acid depletion on proliferative capability and metabolism of HSPC population extracted from day 7 expansion cultures under low O_2_. (**a**) Compares the growth of HSPCs treated with different culture conditions (*N =* 3; 3 wells/condition/experiment). (**b**) Displays the effect of Valine-deprived media on the metabolic potential of HSPCs under baseline and stressed conditions (*N =* 3; 3 wells/condition/experiment). Error bars represent ±SEM. ANOVA and multiple comparison test applied.

We decided to investigate the effect of valine-depletion on the short-term primary HSPCs under low O_2_ conditions, to determine if this led to a reduction in their ability to proliferate. Initially, HSPCs were cultured in StemSpan (SSpan)^TM^ (special medium for in vitro culture and expansion of HSPCs isolated from human, mouse and other species when combined with appropriate growth factors and supplements) and custom-made SSpan^TM^ (+/−AAs) to evaluate their proliferative capacity. HSPCs cultured in custom-made Val-depleted SSpan showed a substantial reduction in the proliferative capability when compared with cells cultured in normal SSpan media or custom-made AA-depleted SSpan medium with all AAs added (Figure 7a). Following this, the metabolic potential of HSPCs after 7 days in culture in media with or without valine under low O_2_ was analysed using Seahorse XF cell energy phenotype assay. HSPCs cultured in Val-deprived media showed significantly lower OCR levels under both baseline and stressed conditions, indicating a reduction in mitochondrial-mediated respiration favoured in proliferating HSPC. Our preliminary data is compatible with the previously published work showing a reduction in number and proliferate capacity, and we show a reduction in their ability to switch to OXPHOS respiration, important in supporting HSPC in a proliferating state. This effect of valine-depletion on HSPC suggests this could possibly affect the incidence of rAML by altering the frequency and maintenance of the target cell population for rAML induction.

## 4. Discussion

The role of oxygen availability and metabolism in the regulation of haematopoietic homeostasis and cellular responses to external oxidative stressors such as IR exposure is increasingly recognised. Many internal and external factors, such as diet and exposure to oxidative stressors influence O_2_ metabolism and so can affect haematopoietic homeostasis and HSPC radio-sensitivity, and so therefore also rAML risk.

In this study we examined both the effects of oxygen levels (ambient normoxia 20.8% vs. low O_2_ 3%) and IR on the growth potential and metabolic phenotype of murine HSPC. Previous in vitro studies used ranging O_2_ levels (1–7%) to revise the influence of hypoxic culture on numerous stem cell micro-environments [60,61,62,63,64,65,66,67,68]. Accordingly, hypoxic culture preserved the redox environment [62], improved cell fitness, differentiation potential, short-term proliferation capability, long-term expansion efficacy and stemness and inhibited senescence.

Our growth curve analyses of HSPCs showed that normoxia increases proliferative capacity in unirradiated HSPC, but also enhances IR-induced reduction in proliferative capacity in irradiated HSPC i.e., increasing O_2_ levels favour HSPC proliferation and differentiation over HSC quiescence as well as increase HSPC radiosensitivity compared to HSPC cultured under low O_2_ conditions. Haematopoietic reconstitution following radiation exposure requires the release of the surviving HSCs from their quiescent state into the G1 phase of the cell cycle. Approximately 60% of surviving HSCs actively cycle for more than 10 months following radiation exposure, with the number of cell divisions per surviving HSC reported to be ten times as high compared with unexposed mice [69]. Our data are therefore compatible with an initial reduction in growth rate due to cell death followed by proliferation to replace the cells so that all the samples had the same number of cells by the end of the short-term culture.

IR stimulates a state of oxidative stress (OS) in cells through direct hydrolysis of water molecules, increasing the expression of inflammatory cytokines and damaging mtDNA. Once activated, these pathways can produce a transient increase in ROS production, eventually leading to HSC exhaustion or pre-leukemic transformation [70,71,72,73]. Normoxia increases HSPC radiosensitivity by enhancing IR-induced ROS production and IR-induced genotoxicity but also increases cell division. Proliferating HSPCs under normoxia are more radio-sensitive, with an increased vulnerability to IR-induced oxidative damage, due to its more open DNA structure and increased DNA content. Conversely, the low O_2_ environment is radio-protective for HSCs by maintaining the quiescent state, reducing both cell division and ROS production.

Upon entry into the G1 phase of the cell cycle, DNA damage is primarily repaired by error prone non-homologous end-joining, potentially promoting the formation of de novo mutations following DNA damage [74]. Additionally, the enhanced replicative stress contributes to premature HSC ageing and induction of HSC premature senescence in a ROS-dependent manner [75], hereby decreasing their DNA repair capacity and rendering them more prone to spontaneous mutations. This hypothesis is further supported by the finding of a common myeloid progenitor-like leukemic stem cell (Lin-Sca1-cKit+CD34+) in a murine rAML model where it was postulated that, amid continuous cycling to reconstitute the CMP population, del2 HSCs acquired additional aberrations, including R235 point mutations of the remaining *Spi1* allele [76]. Increased mutation rate with age might also play a role in the acquisition of secondary point mutations, explaining the relatively long latency between exposure and rAML presentation in mice. In addition, Spi1 directly regulates the HSC cell cycle machinery by inhibition of cell cycle activators and induction of cell cycle inhibitors: loss of Spi1 autoregulation dysregulated the balanced cell cycle regulation, leading to excessive proliferation [77,78], increasing the acquisition of point mutations and eventually resulting in either exhaustion of the HSC pool or leukaemia.

Many diseases including cancers have been shown to involve some form of mitochondrial dysfunction, therefore proper evaluation of mitochondrial function is critical to understand its role in health and pathology [79,80,81,82]. Here we reported an increased mitochondrial-ATP production (OXPHOS) under IR exposure and that normoxia caused HSPCs to acquire more aerobic phenotype, whereas HSPCs under low O_2_ favoured anaerobic glycolysis, which is known to be critical for stemness and quiescence [3,9,12,13,14,15]. Multiple groups reported that IR exposure augments NADPH-oxidase activity and mitochondria dependent-ROS production with reduced HIF-1a levels, triggering cell cycle entry and HSC proliferation [32,83]. It is also acknowledged that OXPHOS is primarily used by leukemic hematopoietic stem cells (LSCs), however, why LSCs favour OXPHOS instead of glycolysis is not fully understood. OXPHOS is a highly efficient way to produce energy and thus, it might be crucial for sustaining LSCs energy need and survival. Furthermore, LSCs can exploit other metabolic events such as amino acid (AA) and fatty acid metabolism (FAO) as an essential source for energy production under metabolic stress environments [84]. According to recent findings, AA and FAO are changed in AML and heavily diminish LSCs survival when inhibited, and thus directly influence OXPHOS maintenance in LSCs [85,86].

Our analysis of several parameters of HSPC mitochondrial function and activity showed that both IR-exposure and normoxia caused a substantial increase in mtDNA, mitochondrial mass and membrane potential. Comparably, a recent study conducted by Qui et al., revealed that human CD34^+^ HSPCs, CD38^−^ HSPCs (CD34^+^CD38^−^), CD90^+^ HSCs and CD49f^+^ HSCs [87] displayed reduced mitochondrial activity (membrane potential [88]) under steady state conditions, signifying the importance of low mitochondrial activity for HSC maintenance [89]. In contrast, AML cells possess significantly higher mitochondrial mass and mtDNA content, showing an upregulated mitochondrial activity to compensate their energy demands under stress conditions [90]. Additionally, increased respiratory activity was reported in AML cells, emphasising the importance of mitochondrial function in both normal haematopoiesis and leukemogenesis [90,91,92,93]. Understanding how metabolism regulates stem cell function in vivo has started to reveal a spectrum of metabolic phenotypes lying between normal homeostasis and cancer. HSCs are metabolically distinct from differentiated cells, possessing unique metabolic properties to maintain the desired quiescent state [47].

Dietary alterations can lead to changes in SC function, influencing the availability of nutrients or regulating hormone levels, growth factors controlling tissue homeostasis and tumour initiation, signalling factors (e.g., insulin and insulin-growth factor), and epigenetic patterns [94,95]. As such it represents an exciting research area for identifying factors influencing leukemogenesis and developing novel treatments capable of mitigating the long-term risks of developing leukaemia following IR exposure. The CBA mouse strain is regarded as the primary model in the study of radiation leukemogenesis [35]. The use of refined models (e.g., genetically modified CBA) has already offered an insight into the mechanisms, allowing the monitoring of preleukemic cells in vivo [36]. This now well characterised model could be used to study the influence of diet or alternate fasting on the sequence of molecular events that occurs during the radiation-induced leukemic clonal evolution and hence, perhaps, lead to the development of protocols reducing the leukaemia induction risk in previously exposed human populations.

Different approaches have been utilized to alter metabolism in vivo [25,94,96,97,98,99,100,101]. CR is known to reduce haematopoietic tumour burden [102] and the incidence of rAML [103] in some mouse models. The exact mechanism is unknown but is thought to involve an effect on the stem cell niche in the bone marrow, reducing the number and availability of the haematopoietic stem/progenitor cells, and therefore the number of potential target cells for rAML. This in turn would then alter the rate of both AML initiation and progression. Studies conducted by Lu et al. [104] revealed that dietary fasting hinders the development of B- and T-cell ALL, but not AML through upregulating leptin-receptor expression (LEPR) and its downstream pathway via the protein PR/SET domain 1 in mouse models of these tumours. Further analysis using a human xenograft model revealed that fasting efficiently hindered the growth of B-ALL [104].

This effect on the stem cell niche is thought to be produced by a change in reactive oxygen species metabolism in the mitochondria of the haematopoietic stem/progenitor cells under CR conditions [105]. Whilst the beneficial effects of CR on whole-body metabolism, including improved insulin and glucose profiles, have been described for decades, recent research has revealed that, on a cellular level, CR affects the same molecular pathways as current biological agents proposed for targeting cancer metabolism. Recent data [106] revealed that CR in mice works synergistically with radiation therapy to target and downregulate several of these pathways and to slow tumour growth.

As stated earlier, valine (and to a lower extent cysteine) are essential for maintaining mouse HSC survival and proliferation [57,58]. In line with these findings, we confirmed that valine-depletion let to a substantial reduction of the proliferative capacity of mouse HSPCs. Our Seahorse XFp analysis revealed that HSPCs cultured in valine-depleted media are metabolically less active both under baseline and stress conditions. Likewise, several groups reported that arginine deprivation has an influence against several cancer cell types including pancreatic, prostate and breast cancers as well as primary AML through altering distinct signalling pathways [107,108,109,110,111]. It can therefore be suggested that valine- or arginine-depletion could possibly reduce the incidence of radiation-induced leukemogenesis, by altering the frequency and maintenance of rAML-target cells within the HSPC compartment.

## 5. Conclusions

In this paper we have presented an analysis of the changes in cellular O_2_ metabolism, mitochondrial activity and function and proliferation potential induced by exposure to ionising radiation in HSPC obtained from CBA/Ca rAML model mice in short-term primary cell culture. Our data shows that IR acts as an oxidative stressor, initially causing cytotoxicity then an increase in proliferation in surviving cells as a response, with a corresponding rise in mitochondrial activity and a switch to OXPHOS respiration. This study provides the basis to further study the effect of dietary changes and other factors which are known to affect HSPC metabolism and haematopoietic homeostasis, which have the potential to act as modifiers of radiation exposure and rAML risk via multiple mechanisms including reduction in rAML target cell populations, curbing over proliferation risk and related prolonged oxidative stress.

## Figures and Tables

**Table 1:** Genomic and mitochondrial primer sequences.

Primer	Sequence
mtND1 Forward	5′-CCCATTCGCGTTATTCTT-3′
mtND1 Reverse	5′-AAGTTGATCGTAACGGAAGC-3′
GAPDH Forward	5′-CAAGGAGTAAGAAACCCTGGACC-3′
GAPDH Reverse	5′-CGAGTTGGGATAGGGCCTCT-3′

## Data Availability

Data is contained within the article.

## Supplementary Materials

The following is available online at https://www.mdpi.com/article/10.3390/antiox11010011/s1, Figure S1a. Simplistic illustration of direct quantitative measurement of H2O2 in biological samples. Figure S1b. Effect of oxygen stress and IR exposure on Hydrogen peroxide levels of HSPC population extracted from day 7 expansion cultures.

## Abbreviations

(AA) Amino Acid; (AML) Acute myeloid leukemia; (ATP) Adenosine-5′-triphosphate; (B-ALL) B-cell Acute Lymphoblastic Leukemia; (BM) Bone marrow; (CR) Caloric restriction; (ECAR) Extracellular acidification rate; (ETC) Electron transport chain; (FAO) Fatty acid metabolism; (FCCP) Carbonyl cyanide-p-trifluoromethox-ypenyl-hydrazon; (GAPDH) Mus musculus glyceraldehyde-3-phosphate dehydrogenase; (GFP) Green Fluorescent protein; (GlycoATP) Glycolytic ATP; (H_2_O_2_) Hydrogen peroxide; (HSCs) Haematopoietic stem cells; (HSPCs) Haematopoietic stem and progenitor cells; (IMDM) Iscove’s Modified Dulbecco’s Media; (IR) Ionising radiation; (LEPR) Leptin-receptor; (LSCs) Leukemic hematopoietic stem cells; (LSK) Lin^-^ Sca-1^+^cKit^+^ cells; (LT-HSCs) Long-term Haematopoietic stem cells; (mitoATP) Mitochondrial ATP; (MPPs) Multi-potent Progenitors; (mtDNA) Mitochondrial DNA; (MtMP) Mitochondrial Membrane potential; (mtND1) Mus musculus mitochondrion; (OCR) Oxygen consumption rate; (OS) Oxidative stress; (OXPHOS) Oxidative phosphorylation; (PBS) Phosphate buffered saline; (PCR) Polymerase chain reaction; (SEM) Standard error of the mean; (rAML) Radiation-induced acute myeloid leukemia; (RNS) Reactive nitrogen species; (ROS) Reactive oxygen species; (SC) Stem cell; (SSpan) StemSpan; (ST-HSCs) Short-term haematopoietic stem cells; (Val-deprived) Valine deprived; (VMT) Threonine.
