# Supplementary material for: Oxidative Stress and X-ray Exposure Levels-Dependent Survival and Metabolic Changes in Murine HSPCs
Source: Antioxidants (Basel). 2021 Dec 22;11(1):11. doi: 10.3390/antiox11010011 (PMC8772903; doi:10.3390/antiox11010011)
Supplement: Supplementary file 1 [file antioxidants-11-00011-s001.zip › antioxidants-1486355-supplementary.pdf]

Supplementary Materials

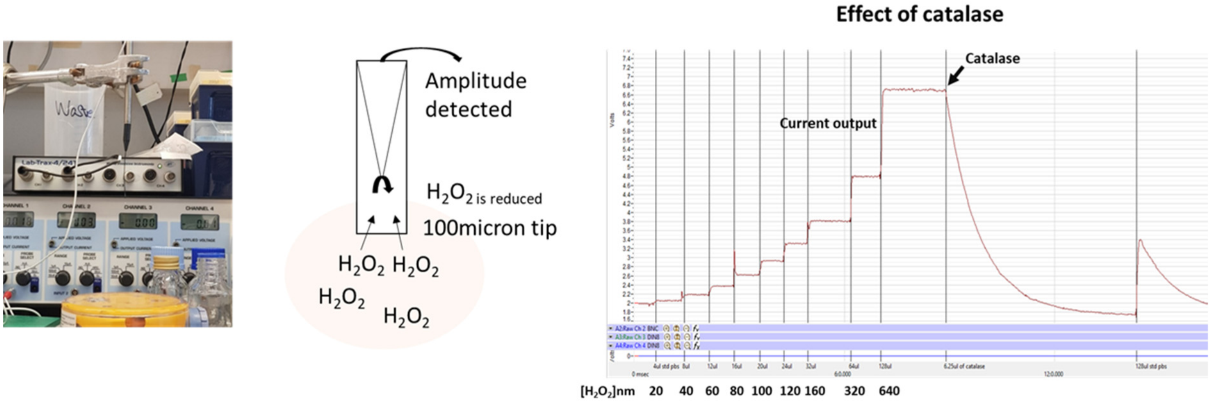

Figure S1a Simplistic illustration of direct quantitative measurement of H_2O_2 in biological samples.

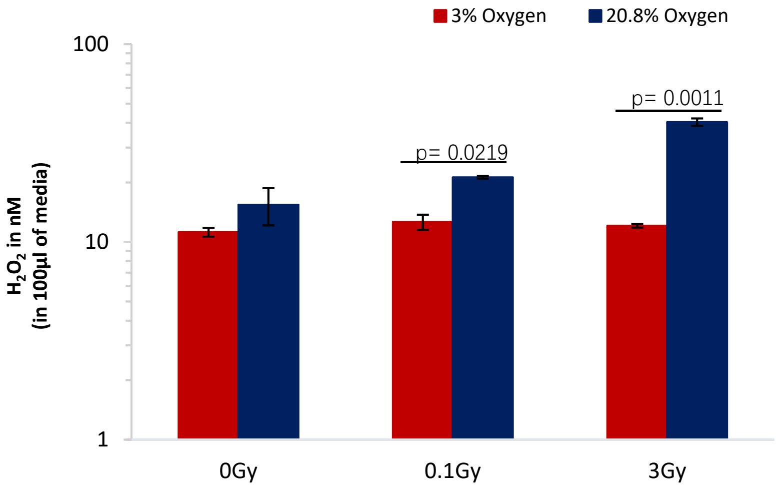

Figure S1b Effect of oxygen stress and IR exposure on Hydrogen peroxide levels of HSPC population extracted from day 7 expansion cultures. HSPCs for each assay is extracted from expansion cultures at day 7. Different oxygen levels and radiation doses compared to assess the changes in H_2O_2 levels. (N:3, Error bars represent ±SEM). Two-way ANOVA and multiple comparison test applied.
